# A suite of global accessibility indicators

**DOI:** 10.1038/s41597-019-0265-5

**Published:** 2019-11-07

**Authors:** Andy Nelson, Daniel J. Weiss, Jacob van Etten, Andrea Cattaneo, Theresa S. McMenomy, Jawoo Koo

**Affiliations:** 1 Faculty of Geo-Information Science and Earth Observation, University of Twente, Enschede, The Netherlands; 2 Malaria Atlas Project, Big Data Institute, University of Oxford, Oxford, UK; 3 Bioversity International, Rome, Italy; 4 Food and Agriculture Organization of the United Nations, Rome, Italy; 5 International Food Policy Research Institute, Washington DC, USA

**Keywords:** Geography, Civil engineering

## Abstract

Good access to resources and opportunities is essential for sustainable development. Improving access, especially in rural areas, requires useful measures of current access to the locations where these resources and opportunities are found. Recent work has developed a global map of travel times to cities with more than 50,000 people in the year 2015. However, the provision of resources and opportunities will differ across the broad spectrum of settlements that range from small towns to megacities, and access to this spectrum of settlement sizes should also be measured. Here we present a suite of nine global travel-time accessibility indicators for the year 2015, at approximately one-kilometre spatial resolution, for a range of settlement size classes. We validated the travel-time estimates against journey times from a Google driving directions application across 1,511 2° × 2° tiles representing 47,812 journeys. We observed very good agreement, though our estimates were more frequently shorter than those from the Google application with a median difference of −13.7 minutes and a median percentage difference of −16.9%.

## Abstract (summary)

**Table:** 

Measurement(s)	travel time to resource • resource accessibility
Technology Type(s)	digital curation • computational modeling technique
Factor Type(s)	population size • location
Sample Characteristic - Environment	city • rural area
Sample Characteristic - Location	Earth (planet)

Machine-accessible metadata file describing the reported data: 10.6084/m9.figshare.9995957

## Background & Summary

Access to the resources, services and opportunities that are concentrated in cities is an important and frequently used indicator for rural development^1^, agricultural productivity^2^, access to markets for both food consumers and food producers^3,4^ and trade^5^. More generally, cities and the transport networks that connect them are essential infrastructure that provide the means for people and products to travel from A to B, thus enabling social and economic interactions and the delivery of basic services such as education and healthcare. Populations with good access generally have greater opportunity for social and economic development, reduced costs and greater levels of interaction, whereas poor access generally means higher costs, fewer opportunities and poorer health and education outcomes.

Inequalities in access can lead to greater social and economic divides^6^. Poorly planned expansions of transport networks can also degrade the natural environment, leading to deforestation and the over exploitation of easily accessed natural resources^7^. On the other hand, well planned improvements in access can lead to better outcomes in rural health, wealth and economic livelihoods whilst limiting environmental impacts^8^.

In 2018, Weiss *et al*.^9^ published a dataset on global travel times to the nearest city of 50,000 or more people for the year 2015 (the baseline for the United Nations Sustainable Development Goals) and demonstrated negative relationships between rural wellbeing and travel time to these cities. This was an update and improvement of a previous dataset for the year 2000^10^ (the baseline for the Millennium Development Goals). The 2015 dataset took advantage of: improved data layers that characterised the size and location of human settlements; more comprehensive information on transport networks and travel speeds; improved environmental layers that characterised off-road speeds, and; computational tools that could account for distance distortions on equirectangular (longitude-latitude) grids when computing travel times.

Both the 2000 and 2015 datasets use travel time as a readily interpretable metric to represent physical access to human settlements. However it is important to differentiate between physical, economic and social access and recognise that having good physical access does not imply similarly good economic and social access. In the case of economic access, a person with good physical access to a human settlement may not have the financial means to use the transport network efficiently. Similarly, members of the same household may face different levels of social access to resources and opportunities in a settlement if they are discouraged or prevented from using them due to cultural norms. Here we use the same travel time metric^9,10^ as a measure of physical access.

The data described here is a further improvement in two respects. Firstly, it takes into account that city size does matter in terms of resources and services provided. One of the limitations of both the year 2000 and year 2015 datasets is that travel time to the nearest city was estimated for all cities of 50,000 or more people. The nearest city could be a megacity with ten million or more people or a small regional city of 50,000 people and anything in between. Within this spectrum of city sizes there are enormous differences in the availability of resources, services and opportunities. Secondly, it considers that there is a substantial share of the population living in settlements that are less than 50,000 people, and these people should not be considered rural by default. The lower limit of 50,000 inhabitants is somewhat arbitrary and access to smaller settlements can be important in many areas. The variation in the provision of resources, services and opportunities across settlements of different size implies that a more nuanced assessment of access across settlements of different sizes will be of interest for regional planners and service providers when considering the impact of investments and policies that affect the level of access to education and health services, markets and job opportunities.

Here we develop a broader range of global accessibility indicators for the year 2015 to represent access to the different resources, services and opportunities that are available in settlements of different population sizes. We made a nine-level stratification of human settlements^11^, using publicly available information on their population^12^ from small settlements of five thousand people or more to megacities of five million or more. For each of the nine levels, the gdistance R package^13,14^ was used to calculate the travel time from any location in the world to the nearest settlement at high spatial resolution. We used existing estimates of the time required to travel across each 30 arc-second pixel of the Earth’s surface using the most likely form of transport over land or water^9^. The output consisted of nine separate accessibility data layers, one per settlement size class, representing the travel time to the nearest settlement.

Validation of the accessibility data layers was conducted on 1,511 2° × 2° tiles, representing different economic resource settings, by comparing travel time estimates between pairs of human settlements from our methodology against driving time estimates from a Google Maps driving directions application.

These accessibility data layers provide essential information on accessibility for sustainable rural development. The layers can be used to identify where inequalities in access exist, to identify where are the opportunities to improve access, and to assess the resulting benefits. Of particular relevance is the ability to differentiate travel time to agglomerations of different sizes as there is growing evidence that impacts of proximity of rural populations to cities and towns of different sizes affects economic and social development in these rural areas^15,16^.

## Methods

The travel time calculations were based on the accumulation of travel time when moving over a regular grid, where every pixel in that grid has a cost or travel time associated with it. This grid is called a friction or cost surface. A least-cost path algorithm was used to find the shortest travel time between a pixel and any nearby settlement (or target) of a given settlement size class. That shortest travel time was recorded in the pixel. The least-cost calculation was repeated for each pixel in the grid to generate a complete map of travel times to the nearest settlement. For processing ease and efficiency, the globe was split into tiles that were eventually mosaiced to produce a single map. The process was repeated for each settlement size class to produce the nine global maps.

### Input datasets

#### Human population settlement data

We used the 2016 version of the Joint Research Centre’s Global Human Settlement Layer (GHSL)^11^ datasets that represent low density urban clusters (LDC) and high density urban centers (HDC) in a raster format (Table 1). LDC are towns, suburbs or small urban areas and are defined as contiguous cells with a population density of at least 300 people/km^2^ and a minimum population of 5,000 inhabitants. HDC are typically cities or large urban areas and are defined as contiguous cells with a population density of at least 1,500 people/km^2^ or a density of built-up infrastructure that is greater than 50%, and a minimum of 50,000 inhabitants. The GHSL data are provided in the GeoTIFF raster format at one km resolution in a World Mollweide projection (coordinate reference system EPSG:54009). Human population estimates for 2015^12^ (Table 1) on a WGS84 30 arc-second raster (coordinate reference system EPSG:4326) were projected to World Mollweide at one km resolution and summed within the LDC and HDC extents to determine the 2015 population per settlement. These settlement areas were then converted to polygons, with settlement ID and population as attributes, and projected to WGS84.

**Table 1:** Input datasets.

Data	Spatial resolution	Values	Projection	Geographic extent	Temporal reference	Format	Source
GHS settlement grid, following the REGIO model 2014 in application to GHSL Landsat and CIESIN GPW v4-multitemporal (1975-1990-2000-2015)	1 km	Pixel values represent the type of settlement	World Mollweide (EPSG:54009)	Global	2015	GeoTIFF	http://data.jrc.ec.europa.eu/dataset/jrc-ghsl-ghs_smod_pop_globe_r2016a
GHS population grid, derived from GPW4, multitemporal (1975, 1990, 2000, 2015)	30 arc seconds	Population counts	WGS84 (EPSG:4326)	Global	2015	GeoTIFF	http://data.europa.eu/89h/jrc-ghsl-ghs_pop_gpw4_globe_r2015a
Friction surface	30 arc seconds	Time required to cross each pixel in minutes per metre	WGS84 (EPSG:4326)	Global	2015	GeoTIFF	https://map.ox.ac.uk/wp-content/uploads/accessibility/friction_surface_2015_v1.0.zip
Land mask based on Global Administrative Boundaries Dataset (GADM) v3.61	30 arc seconds	Binary mask of land/no land	WGS84 (EPSG:4326)	Global	NA	GeoTIFF	https://gadm.org/download_world.html

There are several settlement hierarchy systems based on population. We did not rely on any one system here, but instead derived minimum population thresholds per settlement class from the characteristics of the LDC and HDC datasets and commonly used city size classes^17^ (Table 2). The largest minimum threshold (class 1) of five million was used as a generous definition of a megacity (often classed as ten million or more) to ensure that there were cities in this class in all regions of the world. One million (class 2) and 500,000 (class 3) are used by the United Nations^17^. 200,000 (class 4) and 100,000 (class 5) were chosen as logical steps between 500,000 and 50,000 (class 6) which is the smallest settlement population in the HDC dataset^11^ and is also the same threshold used in the two global accessibility maps^9,10^. Likewise, 20,000 (class 7) and 10,000 (class 8) were chosen as logical steps between 50,000 and 5,000 (class 9) which is the smallest settlement population in the LDC dataset^11^. Table 2 shows that each settlement class contains between 300 million and one billion people. Over 1.3 billion people live in settlements that Weiss *et al*.^9^ simply consider as rural. Also 1 billion people live in cities between 50,000 and 200,000, which for better or for worse, is quite different from living in a city of a million or more.

**Table 2:** Settlement classes, their population thresholds and characteristics.

Settlement class	Minimum population threshold (>=)	Maximum population threshold (<)	Number of settlements in 2015	Sum of population in 2015 in these settlements	Source of settlement data
1	5,000,000	50,000,000	79	941,207,809	HDC
2	1,000,000	5,000,000	421	851,153,118	HDC
3	500,000	1,000,000	581	400,180,511	HDC
4	200,000	500,000	2,096	630,823,940	HDC
5	100,000	200,000	3,694	515,557,120	HDC
6	50,000	100,000	6,973	484,166,417	HDC
7	20,000	50,000	20,457	628,095,955	LDC
8	10,000	20,000	29,286	410,631,333	LDC
9	5,000	10,000	45,795	322,797,326	LDC

The rationale for not adopting a hierarchy system is that there is no single preferred hierarchy to define access; a hierarchy needs to be tailored to the research questions being addressed. So, for example, research on daily commuting from rural areas may decide to rely on proximity being below a given travel time threshold to cities of 50,000 or more. From a different angle, such as research on access to emergency health services options, prioritization may be driven by trade-offs between travel time and the size of the urban centre as a proxy for quality of health care. The nine accessibility data layers provided here will allow users to make their own choices on how to prioritize access based on travel time and characteristics of interest in urban settlements.

#### Travel time data

Estimating travel time from any location on the Earth’s surface to the nearest settlement requires a cost surface that estimates the time required to cross each pixel in the surface. A raster based model permits movement across off-network areas that would not be feasible in a vector based network model. An existing global cost (friction) surface for year 2015 at 30 arc-seconds resolution^9^ was used in this analysis (Table 1). The pixel values are minutes per metre and the data format is GeoTIFF.

The friction surface incorporated the best available information on transport networks (road, rail, river, canal and sea lanes) and travel speeds across them, the characteristics of off-network areas (landcover, slope and elevation) and typical walking speeds across them, and the time required to cross national borders. The friction surface merges all this information into one layer where the resulting pixel value is the fastest travel speed from all the inputs (thus a pixel containing a road and a river will assume that the road crosses over the river and the pixel value will represent the time required to cross that pixel by road). The process of generating the friction surface is fully described in Weiss *et al*.^9^.

#### Land mask

The computation of travel times was done for all land and sea pixels globally between 85°N and 60°S. The final travel time surfaces were masked to show only land and inland water areas. A 30 arc-second mask was derived from the Shapefile version of the Global Administrative Boundaries Dataset (GADM) v3.6^18^ (Table 1).

#### Computation of travel times

The travel time calculations require point locations representing the start and end points of the journey. The start locations were the centres of each pixel in the cost surface. The end locations were regularly spaced points on the boundaries of the human settlement polygons. Thus, each human settlement has multiple points representing its location and is effectively a polygon within the model, which is a more accurate representation of an urban area than a single point. Our calculations represent the travel time from a given location to the nearest point on the boundary of the settlement. The alternative, calculating travel time to the centre of each settlement, seems attractive, but settlement centres are not defined in the GHSL datasets, generating centroids would mean an arbitrary definition of the centre of the settlement, and concave polygons can have centroids outside their boundaries.

#### Processing zones

**Fig. 1:**
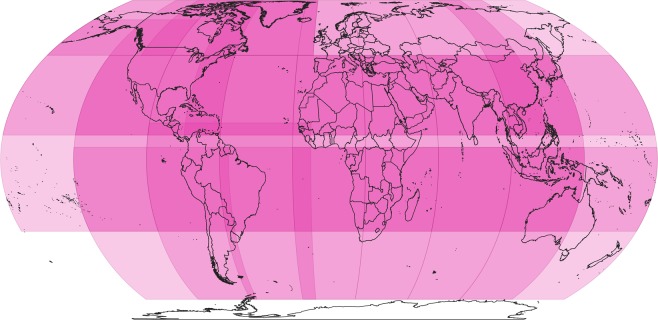
The overlapping processing zones. The map shows the 25 overlapping zones that were used for processing. Another 11 zones were generated that cross the antemeridian (not shown). Country boundaries are from GADM v3.6.

All travel time processing was done in R using the gdistance package^13^. Since gdistance uses functions that cannot operate on huge rasters, the processing was performed zone by zone. The maximum zone size for processing was estimated at around 40° × 40° degrees when using 30 arc-second rasters. These zones need a substantial overlap, as much as 20°, between neighbouring zones to avoid artefacts when calculating the shortest travel time between a pixel and the nearest target. In a process of trial and error, 25 overlapping zones (Fig. 1) of varying sizes were generated for processing and a further 11 zones were generated with all input data re-centred on the antemeridian (180°E or W) to avoid any discontinuities when calculating travel times across the antemeridian.

#### Processing steps

All processing was done in R^19^ (v3.3.3) using RStudio (v1.0.143) on a 32 core server running Windows Server 2012 R2 Standard Edition with 512Gb of RAM. The processing relied on the raster^20^ (v2.6-7) gdistance13 (v 1.2-2) and rgdal^21^ (v1.2-18) packages. The following steps were followed to generate each of the nine accessibility layers, where all processing was done on 30 arc-second resolution rasters with global extent.

1. Clip the friction surface and human settlements (targets) to the spatial extent of each zone.
2. For each zone:

- Generate a transition matrix based on travel in eight directions from each pixel in the friction surface. The transition values are represented by graph connections between neighbouring pixels. The transition value can be computed as the mean of the neighbouring pixel values. However, gdistance expects conductance values instead of resistance values. In this case the pixel value (time to cross one metre) is a resistance value. The conductance was calculated as 1.0/resistance. The transition matrix can be generated using travel in four (orthogonal or von Neumann neighbourhoods), eight (four orthogonal and four diagonal or Moore neighbourhoods) or 16 directions (visualised as a combination of the king and knight moves in chess). We chose eight since it is the most common neighbourhood connection for grids in GIS software and since it was used in the 2015 dataset^9^. Generating the transition matrix is the most memory-intensive and slowest part of the process. To reduce processing time, the transition matrix for each zone was saved to a file which could then be used for all nine accessibility layers.

b. Correct the transition matrix to account for map distortion, as well as for diagonal connections between grid cells. The transition matrix considers eight possible directions of movement from a pixel, however, diagonal neighbours are more remote from each other than orthogonal neighbours and this needs to be corrected. Another correction is needed when working in geographic coordinate systems (e.g. EPSG:4326) since West-East connections become shorter towards the poles, as the meridians approach each other. Both types of distortion can be corrected by dividing each conductance matrix value by the great circle distance between pixel centres.

c. Compute the accumulated cost to travel from any pixel in the zone to the nearest target in the zone and save as a raster.

3. Mosaic the accumulated cost rasters into a single global raster using a minimum function where there are overlapping pixels between rasters. The large overlap between zones ensured that we correctly matched a pixel to its nearest target.
4. Clip the global raster to the land mask and save the output as an integer GeoTIFF at 30 arc-seconds resolution.

## Data Records

**Fig. 2:**
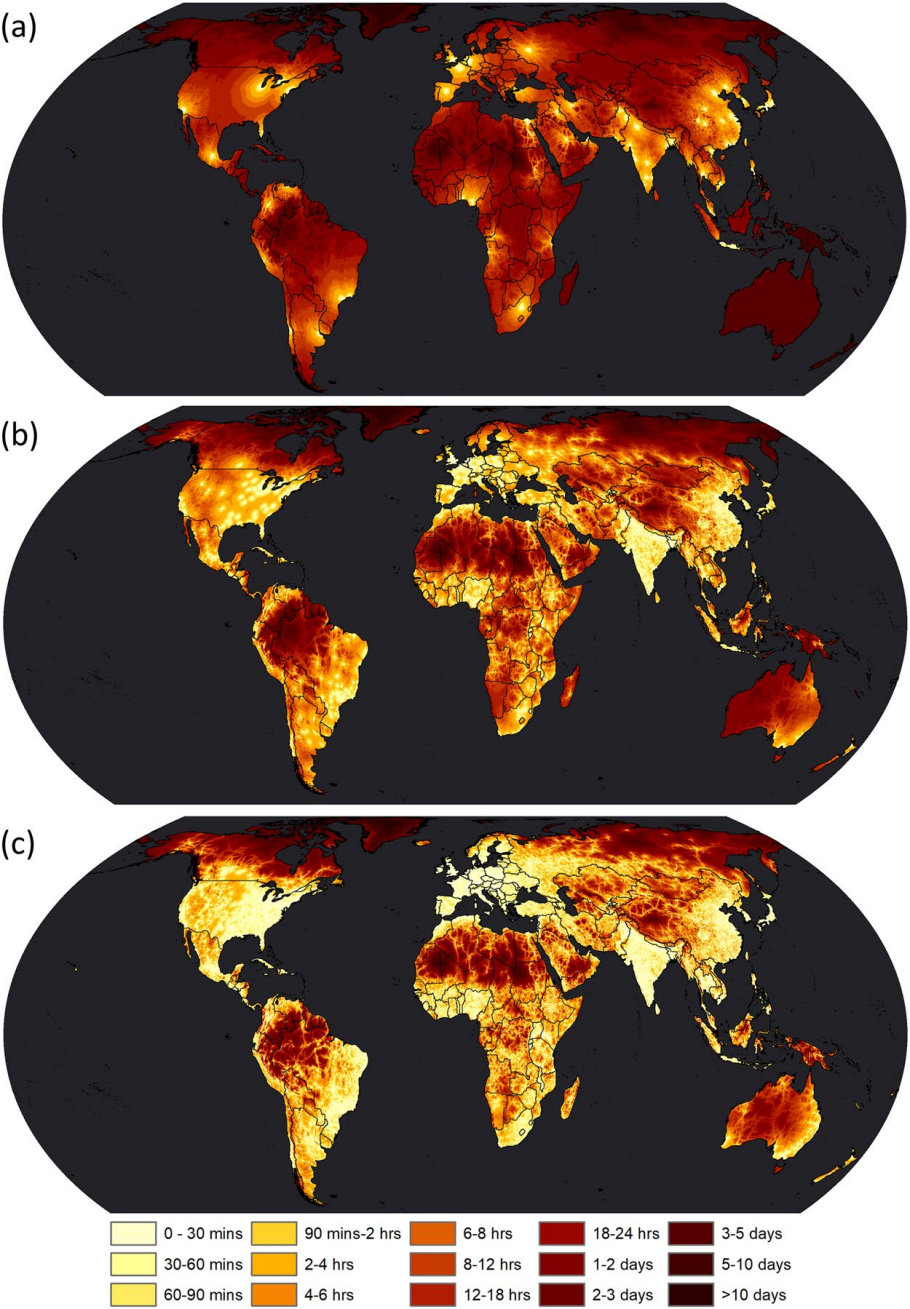
Global accessibility layers. Maps show travel time to the nearest human settlement in the year 2015 for three of the nine accessibility layers, (**a**) for settlement class 1 (>=5,000,000 and <50,000,000 people), (**b**) for settlement class 5 (>=100,000 and <200,000 people), and **(c**) for settlement class 9 (>=5,000 and <10,000 people). Country boundaries are from GADM v3.6.

The nine accessibility layers are available at the figshare repository with appropriate metadata on format, temporal resolution and spatial extent^22^. Each accessibility layer is a 30 arc-second resolution raster in WGS84 (coordinate reference system EPSG:4326) projection with a bounding box of 85°N, 180°E, 60°S and 180°W. The format is single band GeoTIFF, 16 bit unsigned integer with 65,535 as the nodata value. The pixel values represent the time in minutes from that pixel to the nearest settlement (Table 3). Travel times are reported for all pixels classified as land or inland water areas. Figure 2 shows the global maps of travel time in minutes to the nearest human settlements for three of the nine layers.

**Table 3:** Output datasets.

Data	Spatial resolution	Values	Projection	Geographic extent	Temporal reference	Format	File name
Travel time to nearest city between 5,000,000 and 50,000,000 people	30 arc seconds	minutes	WGS84 (EPSG:4326)	Global	2015	GeoTIFF	travel_time_to_cities_1.tif
Travel time to nearest city between 1,000,000 and 5,000,000 people	30 arc seconds	minutes	WGS84 (EPSG:4326)	Global	2015	GeoTIFF	travel_time_to_cities_2.tif
Travel time to nearest city between 500,000 and 1,000,000 people	30 arc seconds	minutes	WGS84 (EPSG:4326)	Global	2015	GeoTIFF	travel_time_to_cities_3.tif
Travel time to nearest city between 200,000 and 500,000 people	30 arc seconds	minutes	WGS84 (EPSG:4326)	Global	2015	GeoTIFF	travel_time_to_cities_4.tif
Travel time to nearest city between 100,000 and 200,000 people	30 arc seconds	minutes	WGS84 (EPSG:4326)	Global	2015	GeoTIFF	travel_time_to_cities_5.tif
Travel time to nearest city between 50,000 and 100,000 people	30 arc seconds	minutes	WGS84 (EPSG:4326)	Global	2015	GeoTIFF	travel_time_to_cities_6.tif
Travel time to nearest city between 20,000 and 50,000 people	30 arc seconds	minutes	WGS84 (EPSG:4326)	Global	2015	GeoTIFF	travel_time_to_cities_7.tif
Travel time to nearest city between 10,000 and 20,000 people	30 arc seconds	minutes	WGS84 (EPSG:4326)	Global	2015	GeoTIFF	travel_time_to_cities_8.tif
Travel time to nearest city between 5,000 and 10,000 people	30 arc seconds	minutes	WGS84 (EPSG:4326)	Global	2015	GeoTIFF	travel_time_to_cities_9.tif

## Technical Validation

We made a spatial validation of the ability of the friction surface to represent travel times. The friction surface is the basis for the travel time estimates in all nine accessibility layers and our expectation is that any bias in the friction layer will have the same impact on each of the accessibility layers. The validation was based on the travel times between pairs of human settlements points from version 1.01 of the Global Rural-Urban Mapping Project^23^ (GRUMP) located on the transport network. We computed travel times between settlements as estimated by the accumulated travel time across the friction surface and compared those to journey times between the same settlements as reported by the Google Maps Platform Distance Matrix API (Application Programming Interface- https://developers.google.com/maps/documentation/distance-matrix).

The Google Maps Platform was selected for validation as it is a widely-accepted means of determining travel time. Unlike our raster-based approach, Google Maps uses a vector-based assessment of movement through a network of roads (each with defined attributes). As such, neither our results nor the Google Map results represent a ‘measured’ (i.e. truth) dataset. It should also be noted that our approach allows for non-road-based travel while the Google Map results do not. In most instances this difference is unimportant because our model will preferentially select routes on roads because they are the fastest means of conveyance. However, in remote areas and wherever settlement travel times to pixels on islands are being calculated it is likely that our approach will involve freer movement off road that the Google Maps API (e.g., the Google Maps approach would include traveling to a port and awaiting a ferry while our model will not). We did not consider variation in journey times due to time of day and day of week, although the API does permit this if a particular departure or arrival time is provided.

We used GRUMP instead of GHSL since our validation method estimates travel time between known locations represented by points (i.e. a gazetteer). GRUMP represents settlement locations as points, whereas as GHSL represents settlements as polygons. The API requires input locations that are situated on or very near to a road, and in our case, they also needed to be within a settlement. These input location could have been derived from GHSL, for example by selecting one point on a GHSL polygon boundary or generating a centroid to represent the settlement, but there are drawbacks to this. The boundary point may not be close to a road, in which case the API will return no data. The centroid would not necessarily represent the centre of the settlement and convex shaped settlements, such as those situated in coastal areas could result in centroids outside the settlement polygon. The GRUMP points were a simpler alternative based on an existing database that required no further data manipulation and they provide a valid set of locations for computing travel time between settlements. Whilst the population and extent of the settlements in the GRUMP dataset will have changed over time, their location remains constant and their population and extent are not relevant for our validation method.

We generated a global coverage of 2° × 2° non-overlapping tiles covering the extent of the friction surface. We identified 1,511 tiles with sufficient human settlements (*n*) to allow the calculation of at least 10 journey times between pairs of settlements [*pairs* = (*n* × (*n-1*))/*2*]. In practice this meant at least five settlements. For each of these tiles, if there were between five and 10 settlements then we selected all settlements. If there were more than 10 settlements, then *i* was calculated as the total number of settlements divided by 10 and we selected every *i*^*th*^ settlement until we reached 10. Travel times between each pair of points was calculated using the accumulated cost function and by the Google API using the mapsapi R package, version 0.4.0^24^. Since travel time was assumed to be the same in both directions, the resulting travel time matrices were symmetrical and we extracted only the lower triangle from the matrix. This resulted in up to 45 pairs in the case of 10 settlements although the number of pairs varied across the tiles. Across the 1,511 tiles, we computed the difference and percentage difference between our travel time estimates and the journey times from the Google API for 47,812 journeys. This provided a spatial assessment of the accumulated travel times between locations across the friction surface.

**Fig. 3:**
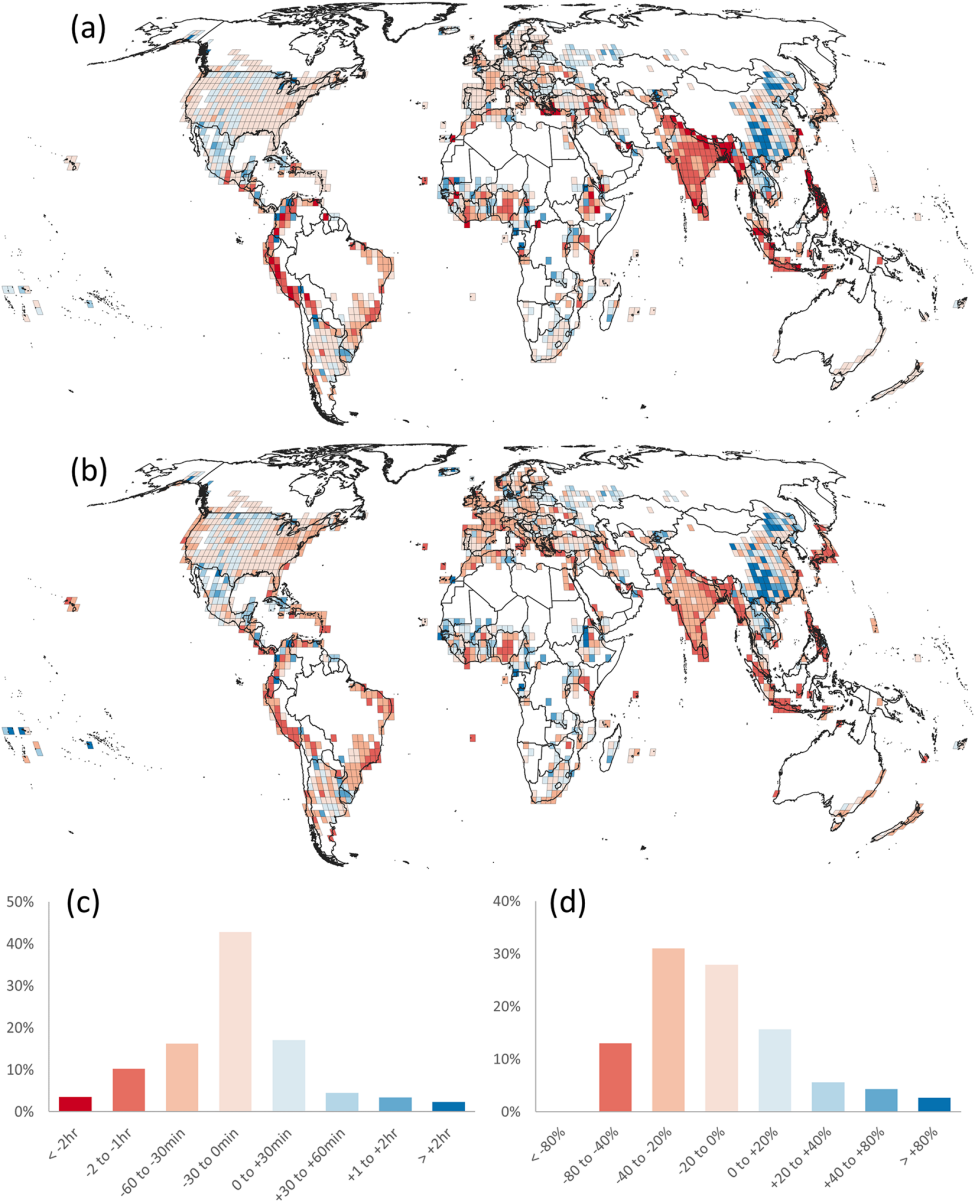
Validation outputs. (**a**) Difference in estimates in minutes (our estimates − Google journey times), (**b**) percentage difference in estimates (100 × [our estimates − Google journey times]/Google journey times), (**c**) histogram of difference in minutes, (**d**) histogram of percentage difference. Panels a and c, and panels b and d have the same colour schemes. Country boundaries are from GADM v3.6.

Our estimated journey times were generally shorter than those from the Google API. Across the tiles, the median journey time from our estimates was 88 minutes within an interquartile range of 48 to 143 minutes while the median journey time estimated by the Google API was 106 minutes within an interquartile range of 61 to 167 minutes. Across all tiles, the differences were skewed to the left and our travel time estimates were shorter than those reported by the Google API in 72% of the tiles. The median difference was −13.7 minutes within an interquartile range of −35.5 to 2.0 minutes (Fig. 3, panel a) while the absolute difference was 30 minutes or less for 60% of the tiles and 60 minutes or less for 80% of the tiles (Fig. 3, panel c). The median percentage difference (Fig. 3, panel b) was −16.9% within an interquartile range of −30.6% to 2.7% while the absolute percentage difference was 20% or less in 43% of the tiles and 40% or less in 80% of the tiles (Fig. 3, panel d).

The spatial information in the validation showed that the smallest differences were in North America, Argentina, Europe, Western Russia, Western Asia, South Africa, Australia and New Zealand. Many of these regions have dense road networks with speed attributes that are likely to be well represented in both the Google database and our friction surface. The largest negative differences, where our travel time estimates were shorter than Google’s, occurred in different regions and likely have different causes. In the Andean region it may be related to over optimistic travel speeds in the friction surface across mountain passes. Slope was used to penalise foot-based travel speeds in the friction surface but not transport network travel speeds. Furthermore, the rasterization of roads to a 30 arc-second resolution raster reduces the sinuosity of the roads and results in a shorter travel time estimate.

Some coastal areas and island archipelagos also had poor fits between the two travel time estimates (Japan, Indonesia and the Philippines. These are areas where driving journey times between settlements on different land masses could not be computed in the Google API resulting in large negative differences for some point pairs.

South Asia, especially India and Bangladesh all showed negative differences, with the largest differences in mountainous and coastal areas. The negative differences here could again be due to the rasterization process where the dense, but not necessarily efficient, transport network would result in spuriously high connectivity in the raster representation compared to the vector-based representation in the Google API. Some regions, such as sub-Saharan Africa had a mixed pattern of negative and positive differences suggesting spatial variability in the quality of transport networks or in the available information on the transportation networks. A large concentration of positive differences was observed in China. This is a known issue due to a lack of transport network information for China in the friction surface meaning that the Google journey times are faster than those estimated across the friction surface.

We also observed a good fit between the two estimates based on linear models between the two travel estimates for each tile, which showed a median adjusted R^2^ of 0.89 within an interquartile range of 0.77 to 0.96 and a median root mean square error of 15.8 minutes within an interquartile range of 7.7 to 31.4 minutes. This overall good fit and the generally low differences between the two travel estimates show that the friction surface represents plausible travel times for mapping accessibility.

The validation results are included with the accessibility layers^22^.

## Usage Notes

The accessibility layers can be visualised and analysed in many Geographic Information Systems or remote sensing software such as QGIS, GRASS, ENVI, ERDAS or ArcMap, and also by statistical and modelling packages such as R or MATLAB. They can also be used in cloud-based tools for geospatial analysis such as Google Earth Engine.

The nine layers represent travel times to human settlements of different population ranges. Two or more layers can be combined into one layer by recording the minimum pixel value across the layers. For example, a map of travel time to the nearest settlement of 5,000 to 50,000 people could be generated by taking the minimum of the three layers that represent the travel time to settlements with populations between 5,000 and 10,000, 10,000 and 20,000 and, 20,000 and 50,000 people.

The accessibility layers also permit user-defined hierarchies that go beyond computing the minimum pixel value across layers. A user-defined complete hierarchy can be generated when the union of all categories adds up to the global population, and the intersection of any two categories is empty. Everything else is up to the user in terms of logical consistency with the problem at hand.

The accessibility layers are relative measures of the ease of access from a given location to the nearest target. While the validation demonstrates that they do correspond to typical journey times, they cannot be taken to represent actual travel times. Errors in the friction surface will be accumulated as part of the accumulative cost function and it is likely that locations that are further away from targets will have greater a divergence from a plausible travel time than those that are closer to the targets. Care should be taken when referring to travel time to the larger cities when the locations of interest are extremely remote, although they will still be plausible representations of relative accessibility. Furthermore, a key assumption of the model is that all journeys will use the fastest mode of transport and take the shortest path.

## Data Availability

The R scripts for generating the suite of accessibility layers and for performing the validation are available at the figshare repository^22^.
